# Test-retest reliability of a new questionnaire on the diet and eating behavior of one year old children

**DOI:** 10.1186/s13104-014-0966-y

**Published:** 2015-01-24

**Authors:** Rachel Kristin Myr, Elling Bere, Nina Cecilie Øverby

**Affiliations:** Department of Public Health, Sports and Nutrition, University of Agder, Serviceboks 422, 4604 Kristiansand, Norway

**Keywords:** Infant/child nutrition, Eating behavior, Breastfeeding, Diet questionnaire, Reliability testing

## Abstract

**Background:**

As part of a sub-study in the ongoing Norwegian RCT ‘Fit for Delivery’, a new questionnaire, using a combination of food frequency, scale, and categorical questions to gather data on the diets and eating patterns of one year olds, was developed and tested for reliability by test-retest.

**Results:**

Of 102 parents recruited to the study, 94 completed both test and retest. Correlation coefficients (Spearman’s r, and/or Cohen’s kappa, where applicable) were high for all categories of question, with a mean value of 0.72 for Spearman’s r for food frequency variables, and a mean value of 0.75 for Cohen’s kappa for non-numeric variables such as breast feeding status, showing very high test-retest reliability.

**Conclusions:**

This newly developed diet and eating habit questionnaire had strong test-retest reliability in a test population similar to the study population, for which it was developed. This indicates that the questionnaire is reliable in this population.

**Electronic supplementary material:**

The online version of this article (doi:10.1186/s13104-014-0966-y) contains supplementary material, which is available to authorized users.

## Background

The strong connection between good nutrition and good health underlies local, national and international initiatives to improve nutrition of pregnant women, infants and young children [1-3]. Because many organ systems are in rapid growth and development in early childhood, diet in this period is especially important [4-8]. Moreover, there is much to suggest that eating habits formed early in life are persistent, and therefore have great potential to affect health throughout life [5,9,10]. The increasing prevalence of overweight and obesity in the population has consequences for reproductive health as well, with increasing intervention in childbirth and concomitant increased risk to mother and child, when the mother is very overweight or the fetus abnormally large [11,12]. It is also possible that factors influencing fetal growth, and thereby the baby’s birthweight, have consequences for the way the individual responds to various nutrients after birth, so that what may be correct diet for one person is pathogenic for another [13]. Recent meta-analyses of interventions to reduce gestational weight gain and thereby reduce adverse outcomes showed that this area of research still contains much uncharted territory [14,15].

‘Fit for Delivery’ (FfD) is a randomized, prospective intervention study in progress, involving nulliparous pregnant women. It seeks to examine the effects of focused nutritional counseling and physical activity on gestational weight gain, birth weight of the babies, and incidence of complications in labor [16]. Women are randomized to receive standard antenatal care, or to receive standard care plus a combination of individual nutrition counseling and physical exercise groups. FfD’s 10 point nutritional component, summarized below, emphasizes both healthy diet and healthy eating habits [17]. Participants in FfD are followed from time of inclusion in mid-pregnancy through the first year of the child’s life. The dietary content and eating habits of participants’ children at age 12 months are examined in general, using a newly developed questionnaire to one of the parents. The questionnaire also aims to look for possible effects of the FfD antenatal intervention on the diets of the children. The purpose of the present study is to assess the test-retest reliability of the newly developed questionnaire.

## Methods

### Design

This study is a test-retest of the questionnaire, in which the respondents answer the same questionnaire on two occasions one to two weeks apart.

### Questionnaire

The questionnaire aims to describe the general diet of 1 year-olds and in addition elicit data on whether the eating patterns of the children differ between the groups in ways that reflect elements of the intervention. The dietary elements of the Fit for Delivery intervention were the ten dietary advices given in pregnancy [17]. These are: 1) Eat regularly, 2) Eat fruits or vegetables as between meal snacks, 3) Drink water 4) Eat vegetables for dinner every day, 5) Eat candy or snack foods only when you truly enjoy it, 6) Don’t eat more than you need to satisfy your hunger 7) Choose small portions of unhealthy foods, 8) Limit your intake of added sugar, 9) Limit your intake of salt 10) Read the nutrition information on labels when you shop.

Such a questionnaire requires detailed information about what and how they eat and drink. In addition to finding out what the child eats, we wanted to know about how eating was handled. The questionnaire includes questions on these topics; background of child’s age and who fills in the questionnaire (3 items), breastmilk and weaning (8 items), intake of beverage (17 items) and food (42 items), portion size of selected beverage (4 items) and foods (8 items), usage of dietary supplements (4 items), meal pattern (13 items), feeding practices (19 items) and questions related to the Fit for Delivery-intervention (4 items) (See questionnaire: Additional file 1).

The questionnaire was constructed using some food frequency questions from the national young child dietary survey ‘Spedkost’ [18], and the new items specific to our purposes, developed to reflect the advice in the FfD intervention. One element in FfD concerns eating to satiety. It appears that the way a child eats and drinks is associated with degree of self-regulation of food intake; for example, Li et al. found that children who were bottle fed in infancy are more likely to drink up the entire portion that is offered them, than children who have not used bottles [19], so we asked about bottle use. We asked about the mealtime setting, too, because it influences how the child eats. Whether the child sits at table with the rest of the family for common meals is not the same as being fed separately at a time when no others are eating [20]. FfD emphasizes planning meals to avoid impulse-driven consumption of less healthy foods so we asked about the circumstances in which such foods were eaten. Because of a trend to eating more often in cafés with young children, we included questions about how often the child ate away from home with a parent and what the child ate on such occasions. There have been no studies published in Norway to date in which such data were collected.

The food frequency questions are simpler than those in Spedkost, in that we ask only about frequency of food intake and not quantity eaten, except for the less desirable foods. We ask for some general information about the child, about breastfeeding status, frequency of use of other beverages, various foods, and dietary supplements such as cod liver oil and vitamins. The very comprehensive Spedkost survey has been carried out twice, in 1999 and 2005, with some modifications between the two surveys, to survey the diets of a representative sample of Norwegian children [18]. By basing our food frequency section on the Spedkost questionnaire, we make it possible to compare the responses of the study population in FfD to those of the population at large from the national surveys.

All food frequency items except those about breastmilk had eight possible responses, ranging from ‘never/less than weekly’ to ‘five or more times daily’. Breastmilk intake was reported as frequencies of day/evening feeds and night feeds in two questions, with seven possible responses for day/evening feeds, from ‘0’ to ‘10 or more’ and four possible responses for night feeds, from ‘0’ to ‘6 times or more’. These were re-coded to give weekly frequencies for ‘breastmilk in day and evening’ and ‘breastmilk at night’ and were analyzed separately. Scale variable questions covered the child’s age in months at weaning from breast, at introduction of other milk and of complementary foods. The child’s willingness to try new foods was expressed on a 10 point Likert scale with 10 being very willing. There are also three questions with four response options ranging from ‘never’ to ‘always’, about how often the child eats the entire portion served, and how often the parent reads nutritional information on the label of their own and of the child’s food. The non-numeric, or categorical, variables covered breastfeeding status (currently breastfed, previously breastfed, never breastfed), and if relevant, primary and secondary reasons for weaning; what drinking vessel the child uses for other beverages now, and if a bottle is used, who holds the bottle, as well as whether the child usually feeds her/himself or is fed, which was asked specifically for each mealtime. In addition there were questions about the source of the child’s food while at day care, and when dining out.

The questionnaire was created using SurveyXact, an interactive program for online questionnaires which tailors the questions presented to each respondent according to their responses to previous questions. Thus, no respondents will see all the questions, nor all the same questions. For example, a parent reporting that the child is breastfed will not be asked about reasons for weaning, and only parents who report that the child is in day care will be asked what the child eats there. The questionnaire was tested by a focus group (n = 5) before the test-retest was carried out. Focus group informants reported that the questionnaire enabled them with few exceptions to describe accurately what the child’s eating habits were and that it was simple to answer.

### Recruitment and study population

To be included in this test-retest, respondents needed to have a child aged 11–16 months at the time of inclusion, have internet access, and give informed, written consent to participate. We expanded the acceptable age range in order to increase the number of eligible participants without including children whose diet was likely to be very different from the FfD population at the time they are asked to complete the questionnaire.

We recruited participants through a combination of strategic posting on pertinent internet sites directed at parents, blogs, including one specifically for fathers, and ‘snowballing’, in which respondents are themselves invited to recruit others from the target group [21]. One paid advertisement was placed, on a website run by the publisher of books for parents of young children. About 90% of participants were recruited through internet forums. Internet access was needed in order to participate, so this recruitment channel did not exclude any otherwise eligible participants. Participants were offered the chance to win a gift certificate worth 1000 Norwegian crowns useable in major stores, as an incentive to participate.

Parents were invited to e-mail the researcher and all subsequent contact was by e-mail, and although participants could phone if they had questions after reading the information about the study, none did so. Interested persons were sent information about the project and a consent form. They returned the consent form by e-mail to the researcher if they wanted to participate. On receipt of the consent form, respondents were registered in the study, assigned a unique identification number, and sent a link to the survey questionnaire.

The theoretical potential population from which our respondents selected themselves consists of parents of approximately 15,000 children in all of Norway, i.e. the total number of children in our target age range during the recruitment period. Only 111 persons contacted the researcher to inquire about the study, and 102 returned the consent forms. Two fathers and 100 mothers, from all regions of Norway, and one expatriate living in Australia, were registered in the study. Two participants, both mothers, did not answer the questionnaire the first time and were lost to follow-up at that point. Of the 100 respondents who answered the first time, 6 did not answer the second time, leaving 94 complete data sets for reliability analysis. There were 45 male and 49 female children.

### Procedure

The online questionnaire allowed respondents to answer at their convenience once they had been assigned an ID number for logging in. It was possible to stop if interrupted and complete it later. It takes approximately 15 minutes to complete the first time. The researcher registered the first date on which each participant had completed the questionnaire. One week after the first response, participants were sent a new e-mail with the link to the questionnaire for the second occasion. Those who did not complete the questionnaire within four days were sent one e-mail reminder. If they did not respond to that, no further action was taken. Sixteen respondents were sent a reminder for the first time, of whom 14 responded. Twenty were sent a reminder for the second time, of whom 14 responded. Two respondents were sent a reminder notice both times. At least one week passed between the first and second responses, with most respondents answering for the second time within 10 days of the first and no respondents having more than 14 days between responses.

### Statistical analysis

Data from the questionnaire were analyzed for correlation using SPSS 19. Spearman’s r and median values are presented for all scale and frequency variables. Scale and frequency variables were also ranked into quartiles for calculating Cohen’s kappa (Tables 1, 2, 3, 4, 5 and 6). For categorical variables only Cohen’s kappa was calculated (Table 7). The two correlational measures were used to show both similarity in the sum of responses to an item on test and retest, and the proportion of individual respondents whose responses were the same on both occasions [22]. According to Field and Cade et al. large correlation coefficients, defined as 0.5 or greater, indicates that the reliability is high [22,23]. The value of Kappa, identifying the strength of agreement, is according to Masson et al. categorized as follows: <0.20: poor, 0.21- 0.40: fair, 0.41- 0.60: moderate, 0.61- 0.80: good, 0.81- 1.00: very good [24].

**Table 1:** **Scale variables: median values reported and correlations coefficients**

	**n** ^**1**^	**Median test**	**Median retest**	**Spearman’s r**	**Cohen’s kappa** ^**2**^
Age at weaning from breast^3^	54	8.5	9	0.99	0.92
Age at intro of other milk^3^	80	8	8	0.97	0.83
Age at intro of complementary feeds^3^	94	5	5	0.91	0.80
Willingness to try new foods^4^	94	10	10	0.86	0.64
Eats up portion^4^	94	2	2	0.68	0.58
Read labels own food^5^	94	2	2	0.82	0.67
Read labels child’s food^5^	94	3	3	0.83	0.72

^1^Number responding varied according to applicability of question. ^2^Scale and frequency variables were ranked into quartile categories for calculating Cohen’s kappa ^3^Ages reported in weeks up to 7, thereafter in whole months. ^4^A 10-point Likert scale on which 10 was “very willing” was used from willingness to try new foods. ^5^Could vary among 4 choices from “never” to “always”. All correlation measures significant at p ≤ 0.001.

**Table 2:** **Meal and meal pattern** ^**1**^ **, median weekly frequencies and correlation coefficients**

	**n**	**Median test**	**Median retest**	**Spearman’s r**	**Cohen’s kappa** ^**2**^
Breakfast	94	7	7	0.58	0.50
Lunch	94	7	7	0.46	0.32
Afternoon between lunch/dinner	94	5	5	0.81	0.56
Dinner	94	7	7	0.33	0.34
After dinner meal	94	7	7	0.68	0.52
Other meal	94	3	4	0.45	0.30
Breakfast with parent	93	7	7	0.62	0.58
Lunch with parent	90	2	2	0.73	0.57
Dinner with parent	94	2	7	0.57	0.42
After dinner meal with parent	85	2	2	0.59	0.47
Bedtime meal with parent	91	5	5	0.66	0.45
Other meal with parent	86	2	2	0.35	0.26
Meals out (café, restaurant)	85	0	0	0.72	0.69

^1^A discrepancy between lists of meals taken, and meals taken with parent, was discovered after questionnaire was launched and will be revised in subsequent versions. ^2^Scale and frequency variables were ranked into quartile categories for calculating Cohen’s kappa. All correlation measures significant at p ≤ 0.001.

**Table 3:** **Beverages, median weekly frequencies and correlation coefficients**

	**n** ^**1**^	**Median test**	**Median retest**	**Spearman’s r**	**Cohen’s kappa** ^**2**^
Breastmilk in daytime^3^	37	17.5	17.5	0.88	0.65
Breastmilk at night^3^	37	14	14	0.85	0.87
Formula milk	94	0	0	0.97	0.62
Full-fat cow’s milk	94	0	0	0.76	0.87
Reduced-fat cow’s milk	94	2	0	0.86	1.00
Cultured cow’s milk	94	0	0	0.94	0.67
Liquid yoghurt	94	0	0	1.00	0.40
Water	94	35	35	0.77	0.61
Child’s beverage	94	0	0	0.70	0.83
Sugared non-fizzy drink	94	0	0	0.73	0.33
Artificially sweet fizzy drink	94	0	0	0.95	1.00
Rruit juice	94	0	0	0.71	0.66
Other beverage	94	0	0	1.00	0.72

Very few respondents using most items on list. Items omitted if not used by any respondents. ^1^Number responding varies according to applicability of question. ^2^Scale and frequency variables were ranked into quartile categories for calculating Cohen’s kappa ^3^Breastmilk frequency in day/eve reported separately from breastmilk at night. All correlation measures significant at p ≤ 0.001.

**Table 4:** **Breakfast and lunch food, median weekly frequencies and correlation coefficients**

	**n**	**Median test**	**Median retest**	**Spearman’s r**	**Cohen’s kappa** ^**1**^
Infant cereal, commercial	94	6	6	0.93	0.74
Infant cereal, homemade	94	0	0	0.82	0.67
Unsweetened musli	94	0	0	0.63	0.54
Fresh fruit	94	7	7	0.72	0.50
Pureed fruit, commercial	94	2	2	0.82	0.59
Fresh vegetables	94	7	7	0.74	0.47
White bread	94	0	0	0.43	0.43
Medium brown bread	94	2	5	0.66	0.35
Whole grain bread	94	7	5	0.70	0.52
Cripsbread/rusks	93	0	0	0.62	0.45
Whey cheese	94	0	0	0.71	0.58
Cheese	94	2	2	0.83	0.54
Liver paste	94	5	5	0.79	0.50
Meat spread/cold cuts	94	0	0	0.61	0.43
Fish spread	94	2	2	0.78	0.55
Jam	94	0	0	0.82	0.75
Butter/margarine	94	5	5	0.84	0.45
Other spread^2^	94	2	2	0.63	0.32

^1^Scale and frequency variables were ranked into quartile categories for calculating Cohen’s kappa.

^2^“Other spread” may refer to an iron-fortified spread omitted from questionnaire but reportedly in widespread use. All correlation measures significant at p ≤ 0.001.

**Table 5:** **Dinner foods, median weekly frequencies and correlation coefficients**

	**n**	**Median test**	**Median retest**	**Spearman’s r**	**Cohen’s kappa** ^**1**^
Sausages	94	0	0	0.54	0.52
Homemade meat dinner	94	2	2	0.70	0.62
Homemade fish dinner	94	2	2	0.62	0.58
Ready made meat dinner	94	0	0	0.57	0.51
Ready made fish dinner	94	0	2	0.55	0.52
Homemade pizza	94	0	0	0.63	0.63
Other dinner foods	94	2	2	0.42	0.42
Rice	94	2	2	0.78	0.76
Potatoes, not crisps	94	2	2	0.80	0.65
Pasta	94	2	2	0.54	0.45
Gravy/cream sauce	94	2	2	0.73	0.62
Vegetables at dinner	94	7	7	0.65	0.51
Commercial baby fish dinner	94	0	0	0.87	0.78
Commercial baby meat dinner	94	2	2	0.85	0.72
Commercial baby vegetables	94	0	0	0.60	0.54

^1^Scale and frequency variables were ranked into quartile categories for calculating Cohen’s kappa. All correlation measures significant at p ≤ 0.001.

**Table 6:** **Snacks, salt, sugar and vitamin supplements, median weekly frequencies and correlation coefficients**

	**n**	**Median test**	**Median retest**	**Spearman’s r**	**Cohen’s kappa** ^**1**^
Fruit or vegetable as snack	94	5	5	0.76	0.42
Plain yoghurt	94	0	0	0.64	0.54
Sweetened yoghurt	94	2	2	0.83	0.65
Ice cream	94	0	0	0.33	0.31
Cookies/crackers	94	0	2	0.84	0.65
Cake/waffle	94	0	0	0.66	0.66
Sweet roll	94	0	0	0.57	0.56
Chocolate	94	0	0	0.69	0.61
Salt snack foods	94	0	0	0.66	0.54
Salt added to child’s food	94	0	0	0.70	0.52
Sugar added to child’s food	94	0	0	0.59	0.58
Cod liver oil^2^	94	0	0	0.92	0.82
Vitamin D drops	94	0	0	0.96	0.88
Other fish oil	94	0	0	0.84	0.66
Multivitamin supplements	94	0	0	0.90	0.69

^1^Scale and frequency variables were ranked into quartile categories for calculating Cohen’s kappa. ^2^Norwegian health authorities recommend daily cod liver oil for everyone over 6 weeks old. All correlation measures significant at p ≤ 0.001.

**Table 7:** **Correlation coefficients, non-numeric variables**

	**n** ^**1**^	**Cohen’s kappa**
Breastfeeding status	94	0.96
Primary reason for weaning	56	0.88
Secondary reason for weaning	56	0.70
Usual drinking vessel	93	0.94
Who holds bottle^2^	11	1.00
Self feed breakfast	93	0.88
Self feed lunch	93	0.68
Self feed dinner	93	0.78
Self feed afternoon	73	0.49
Self feed evening	91	0.71
Self feed other meals	85	0.35
What child eats when dining out	20	0.91
Food source in day care	94	0.87
What foods sent to day care	33	0.40

^1^number responding, n, varies according to applicability of question.

^2^induded in table despite small number of respondents because of 100% correlation between test and retest. There were 2–4 possible responses for all items except reasons for weaning, for which there were 16 choices each for primary and secondary reasons. All correlation measures significant to p ≤ 0.001.

### Ethics

The study was approved by the Regional Committees for Medical and Health research ethics, Norway.

## Results

Correlation coefficients were high or very high for most questions in the questionnaire (Tables 1, 2, 3, 4, 5, 6 and 7). Table 8 shows the mean correlation coefficients, Spearman’s r and/or Cohen’s kappa, for each category of variables measured. Mean value of 0.72 for Spearman’s r for numeric variables, and a mean value of 0.75 for Cohen’s kappa for non-numeric variables, showing very high test-retest reliability. Tables 1, 2, 3, 4, 5, 6 and 7 show median values for test and retest, where relevant, and correlation coefficients for each item shown, grouped by category.

**Table 8:** **Mean correlation coefficients for variables, by category**

**Variable category (number of questions)**	**Mean Spearman’s r**	**SD**	**Mean Cohen’s kappa** ^**1**^	**SD**
Non-numeric variables^2^ (14)			0.75	0.21
Scale variables^3^ (7)	0.86	0.10	0.74	0.12
Food frequency, all items (63)	0.72	0.17	0.59	0.18
*Beverages (17)*	0.75	0.20	0.64	0.23
*Breakfast/lunch foods (18)*	0.70	0.13	0.51	0.13
*Dinner foods (15)*	0.65	0.13	0.59	0.11
*Desserts/snacks (9)*	0.75	0.21	0.68	0.22
*Supplements (4)*	0.90	0.05	0.76	0.10
Meal pattern^4^ (13)	0.58	0.15	0.44	0.11

^1^Scale and frequency variables were ranked into quartile categories for calculating Cohen’s kappa. ^2^breastfeeding status, reasons for breastfeeding cessation, whether the child feeds her/himself and how the child drinks liquids. ^3^age in months at weaning from breast, introduction of other milks and complementary foods; relative frequency of reading labels on food. ^4^weekly frequencies of foods, daily meals, meals eaten together with at least one parent, and meals out. SD included to show central tendency. All correlation measures significant at p ≤ 0.001.

In the non-numeric variable category (Table 7), which contained most of the new items developed specifically for this study, none had Cohen’s kappa values below 0.35 and 11 of the 14 were above 0.67, one even reaching 1, though there were only 11 respondents who were presented with that question. The question was about who held the bottle if the child used one, and the responses were identical both times. It is included despite the low number because the responses were entirely consistent from test to retest.

High correlation coefficients were obtained for the scale variables as well (Table 1). This category contained items about dietary milestones and personal characteristics, like age of weaning from breast and age at introduction of complementary foods.

The lowest correlation coefficients we found were in the meal pattern category, where 4 of 13 Spearman’s r were below 0.5, though none were below 0.33 (Table 2). Tables 3, 4, 5 and 6 show results for the food frequency questions. Median values of zero occurred when very few of the respondents used a particular item. If all respondents reported never using a particular food, the item was omitted from the tables. Spearman’s r values were consistently high for food frequency items.

## Discussion

Our results showing strong test-retest reliability indicate that the questionnaire works well, and it is well accepted by the target group. The correlations presented in our study are in line with the best presented in a new review on reliability on dietary questionnaires in this age group [25].

The highest correlation coefficients were for scale variables (e.g. age at introduction of other foods, breastfeeding status, and degree of self-feeding). Feeding milestones seemed to be clearly remembered by respondents as significant even when they took place many months before answering the questionnaire. The lowest correlation coefficients, though still indicative of moderate to strong correlation, were those having to do with the most ambiguous variables, those using the word ‘other’ (e.g. other beverage, other spread for bread, other meals). This can be because each respondent will interpret the variable differently.

Further we will consider issues which may have biased the results. The diet of young children changes rapidly over the period we were interested in, so we needed to be able to ask our test respondents as close to the child’s first birthday as possible, as that is when FfD participants answer it. We used a shorter test-retest interval than is generally used in reliability testing [26,27]. We wanted to minimize the effects of actual changes in the children’s diets during the study period. At the age of 12 months there are several changes happening in the child’s diet. The national dietary guidelines recommends breastfeeding until at least 12 months of age [28], and many children are no longer breast fed from this age [18]. Further the guideline opens for introducing cow’s milk in the diet from 12 months of age [27]. Since most Norwegian mothers ends their maternity leave at this time, and the children have other day care, the diet of the child changes also for that reason [18]. With a short time interval as in this study, there is however a risk that participants remember the questionnaire so well that they respond based on their previous responses rather than considering the questions individually and responding based on the child’s current diet, and we are unable to control for this [23].

We have tested only the reproducibility of the questionnaire between two occasions for responding (reliability), not validity, which is the correlation between responses to the questionnaire and other measurement methods such as food diaries, for reporting the child’s diet and eating habits. We cannot control for the discrepancy between what parents report on a questionnaire and what the child actually eats, but we believe this effect would be similar in all groups using the questionnaire. All questionnaire-based research is fraught with the risk of this kind of error [23,29]. The actual responses to our questionnaire are consistent with what is known about the diet of young children in Norway [18].

Of more concern for bias is that our respondents may have been more interested in child nutrition than average, in that they responded to online notices about research on the subject. They were offered the chance to win a gift certificate worth 1000 Norwegian crowns, useable in major stores, as an incentive to participate, but this information was not emphasized on the recruitment poster as strongly as the nutritional focus of the study. Comments from the e-mails they sent requesting information about participation confirmed their interest for childrens’ diet. This could lead to inflated correlation coefficient values, in that our respondents may pay more attention to the child’s diet than is typical.

In some settings, online questionnaires might disproportionally exclude segments of the population, reducing the generalizability of conclusions. In 2011, over 97% of Norwegians between the ages of 16 and 44 years had internet access by home computer, 90% of these used the internet daily for 1.5-2 hours, and the average number of computers per household was more than two. Daily internet use from home is positively associated with length of education and with having paid employment, but average daily time on line was over an hour in all groups of the population aged 9 to 66 years [30].

We did not have permission to collect demographic data on the families in our study. The consent form included a blank for the name of the community in which they resided and from this we could see that they came from all regions of the country, but these data were not part of the study. Our only exclusion criteria were child’s age outside the range we sought, not reading Norwegian, not having access to the online questionnaire, and not consenting to participate, while FfD includes nulliparous women in a clearly defined geographic area, with a number of other medical exclusion criteria which for our purposes were irrelevant. Since we used social networking websites in which respondents recruited others in their networks, there is a risk that our respondents are more homogeneous than a random sample of parents from the birth cohort in a way that affected their responses to the questionnaire.

We included no questions about the child’s birth weight, current size, or health status, because those data are already collected elsewhere on the children in FfD, the actual data coming from the child’s health record at the well-child center she or he attends. If our questionnaire is to be used to assess dietary habits in another context, such items would have to be added.

The questionnaire needed to be complex enough to capture issues of relevance in sufficient detail, but short enough to be filled in within a narrow time frame by the parent of a one year old. It needed to be clear and unambiguous for the respondents, so that they understood the questions in the same way on both occasions for replying. It also needed to be interesting enough to motivate them to complete it. The low attrition rate (6%) between test and retest responses suggests that the questionnaire was simple to use, and that respondents found it relevant [27]. In our sample, very few respondents were presented with the more detailed questions on portion size for sweets and snack foods because they reported that the child never ate these foods at all, so we do not know how well accepted the questionnaire might have been in its longest form.

## Conclusion

This newly developed diet and eating habit questionnaire had strong test-retest reliability in a test population similar to the study population, for which it was developed. This indicates that the questionnaire is reliable in this population.. In particular, the new items developed specifically for FfD, the study in which the questionnaire is being used, had very high reproducibility. This will strengthen the conclusions reached from data in FfD’s follow-up component.

## Additional file

Additional file 1:
**Questionnaire survey of the diet of 12 month old children.**

## Abbreviation

FfD: Fit for delivery

## References

[1] WHO. Infant and young child feeding: model chapter for textbooks for medical students and allied health professionals. 2009. http://www.who.int/nutrition/publications/infantfeeding/9789241597494/en/ [Accessed 09.05.2012]. 23905206

[2] WHO/UNICEF. Global strategy for infant and young child feeding. Geneva: World Health Organization; 2003. http://www.who.int/nutrition/publications/infantfeeding/9241562218/en/index.html [Accessed 06.07.2013].

[3] Karanja N, Lutz T, Ritenbaugh C, Maupome G, Jones J, Becker T (2010). The TOTS community intervention to prevent overweight in American Indian toddlers beginning at birth: A feasibility and efficacy study. J Commun Health.

[4] Scerri C, Savona-Ventura C (2010). Early metabolic imprinting as a determinant of childhood obesity. Int J Diabetes Mellit.

[5] Monasta L, Batty GD, Catteneo A, Lutje V, Ronfani L, Van Lenthe FJ (2010). Early-life determinants of overweight and obesity: a review of systematic reviews. Obes Rev.

[6] Dyer J, Rosenfeld C (2011). Metabolic imprinting by prenatal, perinatal, and postnatal overnutrition: a review. Semin Reprod Med.

[7] Barker D, Osmond C, Kajantie E, Eriksson J (2009). Growth and chronic disease: findings in the Helsinki Birth Cohort. Ann Hum Biol.

[8] Moor V, Davies M (2001). Early life influences on later health: the role of nutrition. Acia Pac J Clin Nutr.

[9] Birch LL (1998). Development of food acceptance patterns in the first years of life. Proc Nutr Soc.

[10] Birch LL, Fisher JO (1998). Development of eating behaviors among children and adolescents. Pediatrics.

[11] Voldner N, Frøslie KF, Haakstad L, Bø K, Henriksen T (2009). Birth complications, overweight, and physical inactivity. Acta Obstet Gynecol Scand.

[12] Poobalan AS, Aucott LS, Gurung T, Smith WC, Bhattacharya S (2009). Obesity as an independent risk factor for elective and emergency caesarean delivery in nulliparous women–systematic review and meta-analysis of cohort studies. Obes Rev.

[13] Gluckman PD, Hanson MA (2004). Living with the past: evolution, development, and patterns of disease. Science.

[14] Gardner B, Wardle J, Poston L, Croker H (2011). Changing diet and physical activity to reduce gestational weight gain: a meta-analysis. Obes Rev.

[15] Thangaratinam S, Rogozińska E, Jolly K, Glinkowski S, Duda W, Borowiack E (2012). Interventions to reduce or prevent obesity in pregnant women: a systematic review. Health Technol Assess.

[16] Sagedal LR, Øverby NC, Lohne-Seiler H, Bere E, Torstveit MK, Henriksen T (2013). Study protocol: fit for delivery - can a lifestyle intervention in pregnancy result in measurable health benefits for mothers and newborns? A randomized controlled trial. BMC Public Health.

[17] Øverby NC, Hillesund ER, Sagedal LR, Vistad I, Bere E. The Fit for Delivery study: rationale for the recommendations and test-retest reliability of a dietary score measuring adherence to 10 specific recommendations for prevention of excess weight gain during pregnancy. Matern Child Nutr, 2012; 13. doi:10.1111/mcn.12026. 10.1111/mcn.12026 PMC6860359 23241065

[18] Øverby NC, Kristiansen AL, Andersen LF, Lande B. Spedkost 12 måneder, National Dietary survey among 12 months old children, Spedkost 2006–2007. Norwegian Directorate of Health, Oslo, 2009 (In Norwegian).

[19] Li R, Fein SB, Grummer-Strawn LM (2010). Do infants fed from bottles lack self-regulation of milk intake compared with directly breastfed infants?. Pediatrics.

[20] Brown A, Lee M (2011). A descriptive study investigating the use and nature of baby-led weaning in a UK sample of mothers. Matern Child Nutr.

[21] Morgan DL, Scannell AU (1998). Planning focus groups.

[22] Field A (2009). Discovering statistics using SPSS: (and sex and drugs and rock ‘n’ roll).

[23] Cade J, Thompson R, Burley V, Warm D (2001). Development, validation and utilisation of food-frequency questionnaires – a review. Public Health Nutr.

[24] Masson LF, McNeill G, Tomany JO, Simpson JA, Peace HS, Wei L (2003). Statistical approaches for assessing the relative validity of a food-frequency questionnaire: use of correlation coefficients and the kappa statistic. Public Health Nutr.

[25] Kolodziejczyk JK, Merchant G, Norman GJ (2012). Reliability and validity of child/adolescent food frequency questionnaires that assess foods and/or food groups. J Pediatr Gastroenterol Nutr.

[26] Andersen LF, Bere E, Kolbjørnsen N, Klepp K-I (2004). Validity and reproducibility of self reported intake of fruit and vegetable among 6th graders. Eur J Clin Nutr.

[27] Bere E, Bjørklund LA (2009). Test-retest reliability of a new self reported comprehensive questionnaire measuring frequencies of different modes of adolescents commuting to school and their parents commuting to work - the ATN questionnaire. Int J Behav Nutr Phys Act.

[28] National nutrition and physical education council. Recommendations for infant feeding, Oslo, 2001 (In Norwegian).

[29] Suskie LA (1996). Questionnaire survey research - what works.

[30] Vaage OF. Norsk mediebarometer 2011. Oslo: Statistisk sentralbyrå; 2012. (In Norwegian).

