# Supplementary material for: Test-retest reliability of a new questionnaire on the diet and eating behavior of one year old children
Source: BMC Res Notes. 2015 Jan 24;8:16. doi: 10.1186/s13104-014-0966-y (PMC4320631; doi:10.1186/s13104-014-0966-y)
Supplement: Additional file 1: — Questionnaire survey of the diet of 12 month old children. [file 13104_2014_966_MOESM1_ESM.docx]

| **Questionnaire survey of the diet of 12 month old children**  Dear participant in Fit for Birth!  Thank you for taking part in this diet survey!  We ask that you read this before answering the questionnaire.  This survey aims to gain information about the diet of your child, who is now about 12 months old. The survey is part of the Fit for Birth study.  We want to know about the child’s usual diet. We realize that diet varies from day to day. Please try as best you can to give an ’average’ of the child’s diet. Please keep the past 14 days in mind as you complete the questionnaire. If the frequencies in the questionnaire don’t fit exactly the way your child eats, use the alternative that most closely resembles it.  Thanks for your help!  The Fit for Birth Team | | 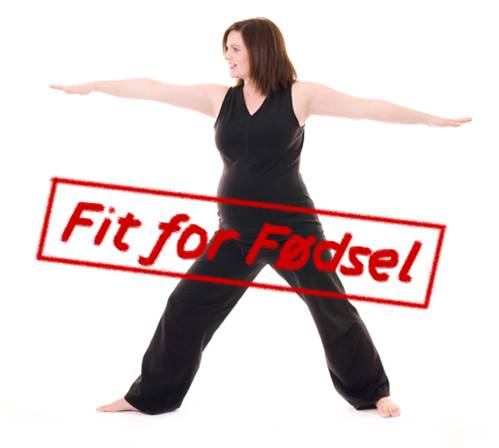<=""> | |
| --- | --- | --- | --- |
| Please enter your participant number here (from letter). | |  |  |
|  | |  |  |

| Date for responding to questionnaire (ddmmyy) |
| --- |
|  |

| BACKGROUND QUESTIONS |
| --- |

| What is the child’s gender? |
| --- |
| 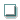Boy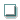Girl |

| Who is responding to the questionnaire? |
| --- |
| 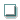Child’s mother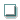Child’s father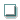Child’s mother and father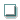Other |

| Who usually cares for the child during the day (weekdays)? |
| --- |
| 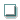Mother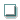Father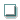Babysitter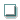Day nursery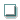Grandparent or other caregiver |

|  |
| --- |

| QUESTIONS ABOUT BREAST MILK |
| --- |

| Does the child receive breast milk now? |
| --- |
| 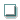Yes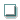No, but has received breastmilk previously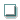No, the child has never had breastmilk |

| How old was the child when it stopped receiving breast milk? Choose one answer. |
| --- |
| 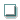1 week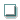2 weeks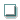3 weeks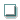4 weeks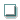5 weeks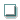6 weeks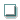7 weeks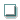2 months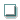3 months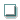4 months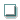5 months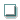6 months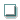7 months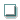8 months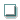9 months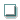10 months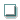11 months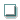12 months |

| What was the primary reason mother did not breastfeed, or has stopped breastfeeding the child? Mark one alternative |
| --- |
| 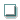Mother did not wish to continue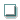Child did not wish to continue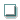Child began biting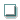Not enough breastmilk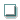Fussiness in the child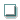Child’s illness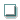Maternal illness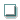Maternal medications (including contraceptives) 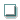New pregnancy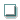Mother returned to work/school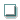Concern for child’s intake of other foods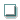Concern for child’s growth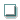Concern for child’s sleep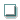Mastitis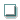Wished to continue but was advised to wean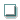Other reasons |

| If mother was advised to wean, did the advice come from: Mark all relevant alternatives |
| --- |
| 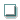Doctor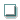Health visitor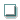Other health professional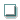Partner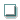Other family member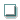Friends |

|  |
| --- |

| What was the next most important reason mother did not breastfeed, or stopped breastfeeding the child? Mark one alternative |
| --- |
| 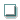Mother did not wish to continue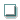Child did not wish to continue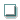Child began biting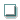Not enough breastmilk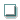Fussiness in the child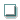Child’s illness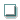Maternal illness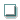Maternal medications (including contraceptives) 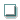New pregnancy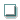Mother returned to work/school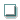Concern for child’s intake of other foods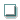Concern for child’s growth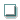Concern for child’s sleep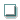Mastitis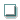Wished to continue but was advised to wean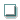Other reasons |

| If mother was advised to wean, did the advice come from: Mark all relevant alternatives |
| --- |
| 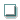Doctor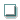Health visitor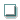Other health professional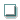Partner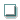Other family member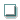Friends |

| How many times does the baby usually receive breast milk now, during the day and evening? |
| --- |
| 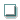0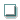1 time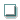2-3 times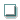4-5 times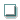6-7 times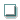8-9 times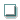10 times or more |

| How many times does the baby usually receive breast milk at night? Include the times the child is fed for comfort or closeness |
| --- |
| 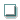0 times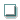1-3 times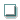4-5 times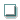6 times or more |

|  | | |
| --- | --- | --- |
| QUESTIONS ABOUT OTHER BEVERAGES | | |
| How old was the child when formula milk or cow’s milk was given for the first time, in addition to or instead of breast milk? Mark one alternative. |  |  |
| 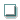1 week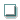2 weeks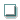3 weeks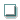4 weeks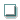5 weeks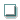6 weeks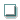7 weeks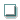2 months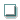3 months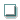4 months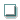5 months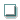6 months7 months8 months9 months10 months11 months12 months |  |  |

| How often does your child usually drink the following beverages now? Mark one choice for each beverage. | | | | | | | | |
| --- | --- | --- | --- | --- | --- | --- | --- | --- |
|  |  |  |  |  |  |  |  |  |
| Infant formula | Never/less than once per week | 1-3 times per week | 4-6 times per week | 1 time per day | 2 times per day | 3 times per day | 4 times per day | 5 or more times per day |
| Full-fat cow’s milk | Never/less than once per week | 1-3 times per week | 4-6 times per week | 1 time per day | 2 times per day | 3 times per day | 4 times per day | 5 or more times per day |
| Reduced fat cow’s milk (light, extra light, skim) | Never/less than once a week | 1-3 times per week | 4-6 times per week | 1 time per day | 2 times per day | 3 times per day | 4 times per day | 5 or more times per day |
| Buttermilk | Never/less than once a week | 1-3 times per week | 4-6 times per week | 1 time per day | 2 times per day | 3 times per day | 4 times per day | 5 or more times per day |
| Chocolate milk | Never/less than once a week | 1-3 times per week | 4-6 times per week | 1 time per day | 2 times per day | 3 times per day | 4 times per day | 5 or more times per day |
| Kefir/yogurt drink | Never/less than once a week | 1-3 times per week | 4-6 times per week | 1 time per day | 2 times per day | 3 times per day | 4 times per day | 5 or more times per day |
| Water | Never/less than once a week | 1-3 times per week | 4-6 times per week | 1 time per day | 2 times per day | 3 times per day | 4 times per day | 5 or more times per day |

| How often does your child usually drink the following beverages now? Mark one choice for each beverage. | | | | | | | | |
| --- | --- | --- | --- | --- | --- | --- | --- | --- |
|  |  |  |  |  |  |  |  |  |
| Child’s beverage | Never/less than once a week | 1-3 times per week | 4-6 times per week | 1 time per day | 2 times per day | 3 times per day | 4 times per day | 5 or more times per day |
| Fruit drink with sugar | Never/less than once a week | 1-3 times per week | 4-6 times per week | 1 time per day | 2 times per day | 3 times per day | 4 times per day | 5 or more times per day |
| Fruit drink with artificial sweetener | Never/less than once a week | 1-3 times per week | 4-6 times per week | 1 time per day | 2 times per day | 3 times per day | 4 times per day | 5 or more times per day |
| Carbonated beverage with sugar | Never/less than once a week | 1-3 times per week | 4-6 times per week | 1 time per day | 2 times per day | 3 times per day | 4 times per day | 5 or more times per day |
| Carbonated beverage with artificial sweetener | Never/less than once a week | 1-3 times per week | 4-6 times per week | 1 time per day | 2 times per day | 3 times per day | 4 times per day | 5 or more times per day |
| Juice | Never/less than once a week | 1-3 times per week | 4-6 times per week | 1 time per day | 2 times per day | 3 times per day | 4 times per day | 5 or more times per day |
| Juice with added sugar | Never/less than once a week | 1-3 times per week | 4-6 times per week | 1 time per day | 2 times per day | 3 times per day | 4 times per day | 5 or more times per day |
| Other beverage | Never/less than once a week | 1-3 times per week | 4-6 times per week | 1 time per day | 2 times per day | 3 times per day | 4 times per day | 5 or more times per day |

| When the child has the following beverages, how large is the serving? Mark one choice for each beverage. | | | | |
| --- | --- | --- | --- | --- |
|  |  |  |  |  |
| Chocolate milk | Half cup (100 ml) | Whole cup (200 ml) | Several cups (more than 200 ml) | Does not get this beverage |
| Fruit drink with sugar | Half cup (100 ml) | Whole cup (200 ml) | Several cups (more than 200 ml) | Does not get this beverage |
| Carbonated beverage with sugar | Half cup (100 ml) | Whole cup (200 ml) | Several cups (more than 200 ml) | Does not get this beverage |
| Fruit juice with or without added sugar | Half cup (100 ml) | Whole cup (200 ml) | Several cups (more than 200 ml) | Does not get this beverage |

| When the child drinks chocolate milk, carbonated, or sweetened beverages, is it usually in connection with: |
| --- |
| Planned special treat, such as Saturday or a café outingUnplannedDoes not drink these beverages |

| How does the child usually drink beverages? |
| --- |
| Cup with spoutOpen cupDrinking bottle with spoutInfant feeding bottle with teat |

| Who normally holds the bottle? |
| --- |
| CaregiverChildEither caregiver or child |

| QUESTIONS ABOUT FOODS | | |
| --- | --- | --- |
| How old was the child when solids were given for the first time? Choose one. |  |  |
| 1 week2 weeks3 weeks4 weeks5 weeks6 weeks7 weeks2 months3 months4 months5 months6 months7 months8 months9 months10 months11 months12 months |  |  |

| How often does the child eat the following foods now? Mark one choice per food. | | | | | | | | |
| --- | --- | --- | --- | --- | --- | --- | --- | --- |
|  |  |  |  |  |  |  |  |  |
| Commercially produced infant cereal | Never/less than once a week | 1-3 times per week | 4-6 times per week | 1 time per day | 2 times per day | 3 times per day | 4 times per day | 5 or more times per day |
| Homemade infant cereal | Never/less than once a week | 1-3 times per week | 4-6 times per week | 1 time per day | 2 times per day | 3 times per day | 4 times per day | 5 or more times per day |
| Müsli (sugar sweetened) | Never/less than once a week | 1-3 times per week | 4-6 times per week | 1 time per day | 2 times per day | 3 times per day | 4 times per day | 5 or more times per day |
| Müsli (unsweetened) | Never/less than once a week | 1-3 times per week | 4-6 times per week | 1 time per day | 2 times per day | 3 times per day | 4 times per day | 5 or more times per day |
| Fresh fruit | Never/less than once a week | 1-3 times per week | 4-6 times per week | 1 time per day | 2 times per day | 3 times per day | 4 times per day | 5 or more times per day |
| Commercially prepared fruit (in a jar, cup or tube) | Never/less than once a week | 1-3 times per week | 4-6 times per week | 1 time per day | 2 times per day | 3 times per day | 4 times per day | 5 or more times per day |
| Fresh vegetables | Never/less than once a week | 1-3 times per week | 4-6 times per week | 1 time per day | 2 times per day | 3 times per day | 4 times per day | 5 or more times per day |

| Bread |
| --- |

| How often does the child eat the following types of bread now? Mark one choice per food. | | | | | | | | |
| --- | --- | --- | --- | --- | --- | --- | --- | --- |
|  |  |  |  |  |  |  |  |  |
| White bread | Never/less than once a week | 1-3 times per week | 4-6 times per week | 1 time per day | 2 times per day | 3 times per day | 4 times per day | 5 or more times per day |
| Medium grain bread | Never/less than once a week | 1-3 times per week | 4-6 times per week | 1 time per day | 2 times per day | 3 times per day | 4 times per day | 5 or more times per day |
| Coarse full-grain bread | Never/less than once a week | 1-3 times per week | 4-6 times per week | 1 time per day | 2 times per day | 3 times per day | 4 times per day | 5 or more times per day |
| Crispbread, rusks | Never/less than once a week | 1-3 times per week | 4-6 times per week | 1 time per day | 2 times per day | 3 times per day | 4 times per day | 5 or more times per day |

| Sandwich spreads | | | | | | | | | | |
| --- | --- | --- | --- | --- | --- | --- | --- | --- | --- | --- |
| How often does the child eat the following foods on bread now? Mark one choice per food. | | | | | | | | |  |  |
|  |  |  |  |  |  |  |  |  |  |  |
| Goat’s (‘brown’) cheese* | Never/less than once a week | 1-3 times per week | 4-6 times per week | 1 time per day | 2 times per day | 3 times per day | 4 times per day | 5 or more times per day |  |  |
| White cheese* | Never/less than once a week | 1-3 times per week | 4-6 times per week | 1 time per day | 2 times per day | 3 times per day | 4 times per day | 5 or more times per day |  |  |
| Liver spread | Never/less than once a week | 1-3 times per week | 4-6 times per week | 1 time per day | 2 times per day | 3 times per day | 4 times per day | 5 or more times per day |  |  |
| Other sandwich meat products | Never/less than once a week | 1-3 times per week | 4-6 times per week | 1 time per day | 2 times per day | 3 times per day | 4 times per day | 5 or more times per day |  |  |
| Fish spreads (mackerel in tomato sauce, caviar)* | Never/less than once a week | 1-3 times per week | 4-6 times per week | 1 time per day | 2 times per day | 3 times per day | 4 times per day | 5 or more times per day |  |  |
| Peanut butter | Never/less than once a week | 1-3 times per week | 4-6 times per week | 1 time per day | 2 times per day | 3 times per day | 4 times per day | 5 or more times per day |  |  |
| Sweet spreads (chocolate spreads, nugatti, or the like) | Never/less than once a week | 1-3 times per week | 4-6 times per week | 1 time per day | 2 times per day | 3 times per day | 4 times per day | 5 or more times per day |  |  |
| Jam/marmalade | Never/less than once a week | 1-3 times per week | 4-6 times per week | 1 time per day | 2 times per day | 3 times per day | 4 times per day | 5 or more times per day |  |  |
| Butter/margarine on the bread | Never/less than once a week | 1-3 times per week | 4-6 times per week | 1 time per day | 2 times per day | 3 times per day | 4 times per day | 5 or more times per day |  |  |
| Other sandwich spreads | Never/less than once a week | 1-3 times per week | 4-6 times per week | 1 time per day | 2 times per day | 3 times per day | 4 times per day | 5 or more times per day |  |  |
| Dinner foods | | | | | | | | | | |

| How often does the child usually eat the following dinner foods now? Mark one choice per food | | | | | | | | |
| --- | --- | --- | --- | --- | --- | --- | --- | --- |
|  |  |  |  |  |  |  |  |  |
| Sausages (hot dogs, wieners and the like) | Never/less than once a week | 1-3 times per week | 4-6 times per week | 1 time per day | 2 times per day | 3 times per day | 4 times per day | 5 or more times per day |
| Homemade meat-based dishes | Never/less than once a week | 1-3 times per week | 4-6 times per week | 1 time per day | 2 times per day | 3 times per day | 4 times per day | 5 or more times per day |
| Homemade fish-based dishes | Never/less than once a week | 1-3 times per week | 4-6 times per week | 1 time per day | 2 times per day | 3 times per day | 4 times per day | 5 or more times per day |
| Commercially prepared meat dishes from an envelope or can (not infant food) | Never/less than once a week | 1-3 times per week | 4-6 times per week | 1 time per day | 2 times per day | 3 times per day | 4 times per day | 5 or more times per day |
| Commercially prepared fish dishes from envelope or can (not infant food) | Never/less than once a week | 1-3 times per week | 4-6 times per week | 1 time per day | 2 times per day | 3 times per day | 4 times per day | 5 or more times per day |
| Homemade pizza | Never/less than once a week | 1-3 times per week | 4-6 times per week | 1 time per day | 2 times per day | 3 times per day | 4 times per day | 5 or more times per day |
| Other dinner foods (soup, pancakes, frozen pizza, rice porridge) | Never/less than once a week | 1-3 times per week | 4-6 times per week | 1 time per day | 2 times per day | 3 times per day | 4 times per day | 5 or more times per day |

| How often does the child usually eat the following dinner foods now? Mark one choice per food | | | | | | | | |
| --- | --- | --- | --- | --- | --- | --- | --- | --- |
|  |  |  |  |  |  |  |  |  |
| Rice | Never/less than once a week | 1-3 times per week | 4-6 times per week | 1 time per day | 2 times per day | 3 times per day | 4 times per day | 5 or more times per day |
| Potatoes | Never/less than once a week | 1-3 times per week | 4-6 times per week | 1 time per day | 2 times per day | 3 times per day | 4 times per day | 5 or more times per day |
| Pasta | Never/less than once a week | 1-3 times per week | 4-6 times per week | 1 time per day | 2 times per day | 3 times per day | 4 times per day | 5 or more times per day |
| Sauce (gravy/white) | Never/less than once a week | 1-3 times per week | 4-6 times per week | 1 time per day | 2 times per day | 3 times per day | 4 times per day | 5 or more times per day |
| Vegetables for dinner, either on their own or in homemade dish with fish or meat | Never/less than once a week | 1-3 times per week | 4-6 times per week | 1 time per day | 2 times per day | 3 times per day | 4 times per day | 5 or more times per day |

| Commercially produced, ready-to-feed baby food |
| --- |

| How often does the child usually eat commercially produced, ready-to-feed baby food now? Mark one choice per food | | | | | | | | |
| --- | --- | --- | --- | --- | --- | --- | --- | --- |
|  |  |  |  |  |  |  |  |  |
| Fish and vegetables | Never/less than once a week | 1-3 times per week | 4-6 times per week | 1 time per day | 2 times per day | 3 times per day | 4 times per day | 5 or more times per day |
| Meat and vegetables | Never/less than once a week | 1-3 times per week | 4-6 times per week | 1 time per day | 2 times per day | 3 times per day | 4 times per day | 5 or more times per day |
| Vegetables only | Never/less than once a week | 1-3 times per week | 4-6 times per week | 1 time per day | 2 times per day | 3 times per day | 4 times per day | 5 or more times per day |

| Other foods |
| --- |

| How often does the child usually eat the following foods now? Mark one choice per food | | | | | | | | |
| --- | --- | --- | --- | --- | --- | --- | --- | --- |
|  |  |  |  |  |  |  |  |  |
| Yogurt without sugar (i.e. plain yoghurt) | Never/less than once a week | 1-3 times per week | 4-6 times per week | 1 time per day | 2 times per day | 3 times per day | 4 times per day | 5 or more times per day |
| Yogurt with added sugar (i.e. fruit yogurt, litago yogurt)* | Never/less than once a week | 1-3 times per week | 4-6 times per week | 1 time per day | 2 times per day | 3 times per day | 4 times per day | 5 or more times per day |
| Ice cream | Never/less than once a week | 1-3 times per week | 4-6 times per week | 1 time per day | 2 times per day | 3 times per day | 4 times per day | 5 or more times per day |
| Pudding/gelatine dessert | Never/less than once a week | 1-3 times per week | 4-6 times per week | 1 time per day | 2 times per day | 3 times per day | 4 times per day | 5 or more times per day |
| Biscuits/cookies | Never/less than once a week | 1-3 times per week | 4-6 times per week | 1 time per day | 2 times per day | 3 times per day | 4 times per day | 5 or more times per day |
| Cakes/waffles | Never/less than once a week | 1-3 times per week | 4-6 times per week | 1 time per day | 2 times per day | 3 times per day | 4 times per day | 5 or more times per day |
| Sweet rolls | Never/less than once a week | 1-3 times per week | 4-6 times per week | 1 time per day | 2 times per day | 3 times per day | 4 times per day | 5 or more times per day |

|  | | | | | | | | | | |
| --- | --- | --- | --- | --- | --- | --- | --- | --- | --- | --- |
| How often does the child usually eat the following foods now?  Mark one choice per food | | | | | | | | |  |  |
|  |  |  |  |  |  |  |  |  |  |  |
| Chocolate | Never/less than once a week | 1-3 times per week | 4-6 times per week | 1 time per day | 2 times per day | 3 times per day | 4 times per day | 5 or more times per day |  |  |
| Candies | Never/less than once a week | 1-3 times per week | 4-6 times per week | 1 time per day | 2 times per day | 3 times per day | 4 times per day | 5 or more times per day |  |  |
| Salty snacks | Never/less than once a week | 1-3 times per week | 4-6 times per week | 1 time per day | 2 times per day | 3 times per day | 4 times per day | 5 or more times per day |  |  |
| Instant noodles (Asian type) | Never/less than once a week | 1-3 times per week | 4-6 times per week | 1 time per day | 2 times per day | 3 times per day | 4 times per day | 5 or more times per day |  |  |

| When the child eats ice cream, what size is the serving? |
| --- |
| Small (less than one scoop)Medium (1-2 scoops)Large (3 or more scoops) Does not eat ice cream |

| When the child eats pudding or gelatine, what size is the serving? |
| --- |
| Small portion (2 tablespoons or less)Medium (3-6 tablespoons)Large (more than 6 tablespoons)Does not eat pudding or gelatine |

| When the child eats cookies/biscuits, how many does s/he eat at a serving? |
| --- |
| 1 or fewer1-3 4 or moreDoes not eat cookies/biscuits |

|  | | |
| --- | --- | --- |
| When the child eats cakes/waffles, how many pieces/hearts* does s/he eat? |  |  |
| A quarter slice or less/1 heart or lessA half slice/2-4 waffle heartsA whole slice or more/a whole waffle or more Does not eat cake/waffles |  |  |

| When the child eats sweet rolls, how much does s/he eat? |
| --- |
| 1/4 roll or less 1/2 rollOne roll or moreDoes not eat boller |

| When the child eats chocolate, how many pieces does the child eat per occasion? |
| --- |
| 1-2 pieces or less3-6 biter7 pieces or moreDoes not eat chocolate |

| When the child eats candy, how many pieces does the child eat per occasion? |
| --- |
| 1-2 pieces or less 3-6 pieces7 pieces or flereDoes not eat candy |

| When the child eats salty snacks, how much does the child eat per occasion? |
| --- |
| Half cup or less One cupMore than one cupDoes not eat salty snacks |

| How often do you salt the child’s food? |
| --- |
| Never/less than once a week1-3 times per week4-6 times per weekOnce a daySeveral times a day |

| How often do you add sugar to the child’s food? |
| --- |
| Never/less than once a week1-3 times per week4-6 times per weekOnce a daySeveral times a day |

|  |
| --- |

| QUESTIONS ABOUT DIETARY SUPPLEMENTS |
| --- |

| How often does the child get Vitamin D or other dietary supplement? | | | | | |
| --- | --- | --- | --- | --- | --- |
|  |  |  |  |  |  |
| Cod liver oil | Never/less than once a week | 1-3 times per week | 4-6 times per week | 1 time per day | 2 or more times per day |
| Vitamin D drops | Never/less than once a week | 1-3 times per week | 4-6 times per week | 1 time per day | 2 or more times per day |
| Other forms of fish oil | Never/less than once a week | 1-3 times per week | 4-6 times per week | 1 time per day | 2 or more times per day |
| Multivitamins (i.e. Sana-sol, Biovit) | Never/less than once a week | 1-3 times per week | 4-6 times per week | 1 time per day | 2 or more times per day |

| QUESTIONS ABOUT MEALS |
| --- |

| How many times per week does the child usually eat the following meals? Mark one choice per meal | | | | | | | | |
| --- | --- | --- | --- | --- | --- | --- | --- | --- |
|  |  |  |  |  |  |  |  |  |
| Breakfast | Never/less than once a week | 1 time per week | 2 times per week | 3 times per week | 4 times per week | 5 times per week | 6 times per week | Daily |
| Lunch | Never/less than once a week | 1 time per week | 2 times per week | 3 times per week | 4 times per week | 5 times per week | 6 times per week | Daily |
| Afternoon snack | Never/less than once a week | 1 time per week | 2 times per week | 3 times per week | 4 times per week | 5 times per week | 6 times per week | Daily |
| Dinner | Never/less than once a week | 1 time per week | 2 times per week | 3 times per week | 4 times per week | 5 times per week | 6 times per week | Daily |
| Meal before bedtime | Never/less than once a week | 1 time per week | 2 times per week | 3 times per week | 4 times per week | 5 times per week | 6 times per week | Daily |
| Other meals/snacks | Never/less than once a week | 1 time per week | 2 times per week | 3 times per week | 4 times per week | 5 times per week | 6 times per week | Daily |

| How often does the child eat fruit or vegetables as a snack? Mark one alternative |
| --- |
| Never/less than once a week1-3 times per week4-6 times per weekDaily Several times daily |

|  |
| --- |

| Does the child bring food from home to day care, or does the day care provide food? |
| --- |
| Brings food from homeFood is provided by day care Has food from home and from day careDoes not attend day care |

| What kind of food does the child bring to day care from home? |
| --- |
| BeverageSandwichDinnerSnack |

| How often does the child eat the following meals at day care? | | | | | | |
| --- | --- | --- | --- | --- | --- | --- |
|  |  |  |  |  |  |  |
| Breakfast | Never/less than once a week | 1 time per week | 2 times per week | 3 times per week | 4 times per week | 5 times per week |
| Lunch | Never/less than once a week | 1 time per week | 2 times per week | 3 times per week | 4 times per week | 5 times per week |
| Dinner | Never/less than once a week | 1 time per week | 2 times per week | 3 times per week | 4 times per week | 5 times per week |
| Snack | Never/less than once a week | 1 time per week | 2 times per week | 3 times per week | 4 times per week | 5 times per week |
| Meal before bedtime | Never/less than once a week | 1 time per week | 2 times per week | 3 times per week | 4 times per week | 5 times per week |

| How often does the child eat the following meals with another family member, i.e. at the same time as at least one adult eats the same meal? | | | | |  |  |
| --- | --- | --- | --- | --- | --- | --- |
|  |  |  |  |  |  |  |
| Breakfast | Never/less than once a week | 1-3 times/week | 4-6 times/week | Daily |  |  |
| Lunch | Never/less than once a week | 1-3 times/week | 4-6 times/week | Daily |  |  |
| Dinner | Never/less than once a week | 1-3 times/week | 4-6 times/week | Daily |  |  |
| Afternoon snack | Never/less than once a week | 1-3 times/week | 4-6 times/week | Daily |  |  |
| Meal before bedtime | Never/less than once a week | 1-3 times/week | 4-6 times/week | Daily |  |  |
| Other meals | Never/less than once a week | 1-3 times/week | 4-6 times/week | Daily |  |  |
|  | | | | | | |

| Is your child usually fed (i.e. an adult holds the spoon or cuts up the food and gives it piece by piece) or does s/he usually feed her/himself (i.e. the child has own plate of food with or without utensils) ? | | |
| --- | --- | --- |
|  | Feeds self | Is fed by someone |
| Breakfast | Feeds self | Is fed by someone |
| Lunch | Feeds self | Is fed by someone |
| Dinner | Feeds self | Is fed by someone |
| Afternoon snack | Feeds self | Is fed by someone |
| Meal before bedtime | Feeds self | Is fed by someone |
| Andre måltider | Feeds self | Is fed by someone |

| How often does your child eat all the food s/he is served at regular meals? |
| --- |
| AlwaysMost of the timeSometimesNever |

| How willing is your child to taste new foods? (On a scale from 1-10, where 1 is ‘unwilling to taste new foods at all’, and 10 is ‘very willing’) |
| --- |
| \| 1 \| 2 \| 3 \| 4 \| 5 \| 6 \| 7 \| 8 \| 9 \| 10 \| \| --- \| --- \| --- \| --- \| --- \| --- \| --- \| --- \| --- \| --- \| \| 1 \| 2 \| 3 \| 4 \| 5 \| 6 \| 7 \| 8 \| 9 \| 10 \| |

| How often does the child eat meals out, in the company of at least one parent (café, restaurant, other outing)? |  |  |
| --- | --- | --- |
| Never/less than once a week1-3 times per week4-6 times per weekDaily |  |  |
|  | | |

| What does the child usually eat when the meal is at a café or restaurant? |
| --- |
| Eats own food from the menu Shares the adult’s food from the menu Eats food from homeOther |

| How often do you read the ingredients on the labels of foods in the store? | | | | |
| --- | --- | --- | --- | --- |
|  |  |  |  |  |
| On food for yourself | Never | Occasionally | Most of the time | Always |
| On food for the child | Never | Occasionally | Most of the time | Always |

| Thank you for completing this questionnaire! |
| --- |
